# How Pastoral Are Pastoral Landscapes? Scavenger Assemblage Structure in Human‐Dominated Landscapes: A Case Study From Mediterranean Pastures

**DOI:** 10.1002/ece3.72839

**Published:** 2026-01-14

**Authors:** Ori Shapira, Ido Izhaki, Shiri Zemah‐Shamir, Dan Malkinson

**Affiliations:** ^1^ Department of Geography and Environmental Studies University of Haifa Haifa Israel; ^2^ Israel Nature and Parks Authority Jerusalem Israel; ^3^ Department of Evolutionary and Environmental Biology University of Haifa Haifa Israel; ^4^ School of Sustainability Reichman University Herzliya Israel; ^5^ Shamir Research Institute University of Haifa Haifa Israel

**Keywords:** carrion ecology, facultative scavengers, pastoralism, PSEM, species interactions

## Abstract

Pastoralism is one of the most common land uses worldwide and has been a fundamental part of Mediterranean ecosystems for thousands of years. We aimed to investigate how this land use influences carrion ecology and species interactions among mammalian facultative scavengers in East Mediterranean habitats. We carried out an in situ “cafeteria” field experiment, placing domesticated ( *Bos taurus* ) versus native ( *Sus scrofa* ) species’ carrion in both pastoral and nonpastoral areas. We monitored scavenger activity using camera traps and analyzed changes in species interactions and carrion preferences between these two habitat types. The average camera encounter rate of the three most common scavenger species was significantly higher in nonpastoral areas than in pastoral ones. Our results also showed a notable preference for boar over cow carrion by foxes ( *Vulpes vulpes* ), while wild boars ( *Sus scrofa* ) and golden jackals ( *Canis aureus* ) exhibited no significant preferences. Using Piecewise Structural Equation Modeling (PSEM), we found no significant interactions among facultative scavengers in pastoral lands, whereas most scavenger species interactions in nonpastoral areas were significant and negative. Our findings indicate a significant, though variable, impact of pastoralism on facultative scavengers. From a conservation standpoint, these results highlight the challenge of maintaining an optimal structure for the mammalian scavenger guild in mixed landscapes.

## Graphical abstract

An in situ field experiment accompanied by camera trapping has shown how pastoralism as a land use affects carrion preferences and species interactions among facultative scavengers.

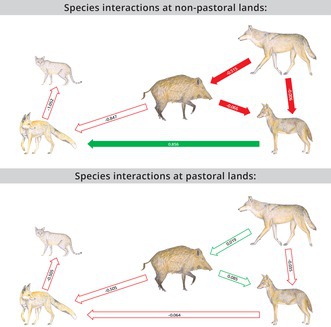

## 1 Introduction

Pastoralism, defined as livestock grazing in natural habitats, is one of the most widespread land uses globally, occupying nearly a quarter of the world's terrestrial surface (Dong 2016). The global livestock biomass, estimated at 0.1 gigatons of carbon (Bar‐On et al. 2018), has far‐reaching ecological consequences, including the replacement of native ungulate populations and their ecological roles across continents (2017; Herrik et al. 2023; Pringle et al. 2023). This broad‐scaled land use makes livestock husbandry an inherent component of many ecosystems, directly or indirectly shaping ecological processes (Yap 2019). In the eastern Mediterranean, particularly northern Israel, cattle grazing is a key driver of landscape structure, notably by opening up dense Mediterranean forests (Dufour‐Dror 2007; Perevolotsky 2005; Schoenbaum et al. 2018). Such “spacing” may facilitate wildlife movement, yet this phenomenon remains insufficiently studied. Adaptive cattle grazing creates a mosaic of habitat patches with varying grazing intensities (Török et al. 2024), and pastoralism is recognized as an important management tool for maintaining biodiversity in Mediterranean habitats (Burke 2010). Despite these recognized effects, the influence of cattle grazing on the structure of local scavenger communities compared to nonpastoral lands is not well understood.

The eastern Mediterranean region, and Israel in particular, has a documented history of livestock domestication and cultivation spanning over 10,000 years (Arbuckle and Hammer 2019; Marom and Bar‐Oz 2013). Evidence suggests that since the Paleolithic era, pastoralism has been a persistent, though fluctuating factor in shaping Mediterranean ecosystems (Burke 2010; Florenzano 2019; Seid et al. 2016).

Pastoralism can alter ecological processes through several mechanisms, notably by enriching habitats with anthropogenic resources. Food supplements such as cattle feed, manure, or hay, used to support livestock nutrition, can have significant evolutionary and ecological impacts, including altering predator–prey dynamics, supporting specific species groups, and reducing community diversity (Ciucci et al. 2020; Oro et al. 2013; Polis et al. 1997; Williams et al. 2022; Magioli et al. 2019). Pastoralism also introduces additional resources in the form of live cattle, which serve as prey for predators, and carcasses, which create spatial hotspots for both obligate and facultative scavengers (Newsome et al. 2024, 2015). The ecological effects of cattle carcasses have been somewhat studied and it was found for example that carcass type can have a stronger effect on scavenger ecology than landscape connectivity (Olson et al. 2016), and that large carcasses like those of cattle are primarily utilized by facultative terrestrial scavengers (Moleón et al. 2015). Carcasses also contribute to the “landscape of fear” for wildlife, underscoring the need to study the less‐explored aspects of carrion ecology (Moleón and Sánchez‐Zapata 2021; Laundré et al. 2010; Redondo‐Gómez et al. 2023). The response of terrestrial facultative scavengers to different carrion types is inconsistent and context‐dependent. To date and to the best of our knowledge, only sparse studies were conducted, to examine specific scavengers preferences when presented with both domestic (cattle) and wild (boar) carcasses. Additionally, the direct effect of pastoralism as a land use and its effects on facultative scavenger dynamics have not been studied in depth, but see Oliva‐Vidal et al. (2022) for an indirect example. Olea et al. (2022), however, studied the direct relationship between pastoralism and scavenging community and documented temporal segregation patterns within a scavenger community in Northern Spain. They demonstrated that subordinate species generally alter their diurnal activity patterns to avoid apex scavengers or, more specifically, that vultures usually have temporal overlaps with corvid species.

Livestock management further supplements ecosystems with protein‐rich feeds, such as poultry litter and cattle feed, yet the use of these resources by wildlife and scavengers remains understudied (but see Bino et al. 2010; Lanszki et al. 2018; Sebastián‐González et al. 2023). For example, it has been suggested that rodents, attracted to stored cattle feed, may increase in abundance near pastoral areas, potentially attracting carnivores that prey on them (Witmer 2022). The combined effects of supplemental feeding and increased prey/carrion availability could influence the abundance, distribution, and behavior of facultative scavengers and medium‐sized carnivores. Additionally, the artificial addition of potable water to pastures, especially in semi‐arid environments, can alter ecological processes by providing critical resources during dry periods, as documented in the Jordanian and Mojave deserts (Attum et al. 2017; Edwards et al. 2017, 2016; Rich et al. 2019).

This study focuses on the terrestrial scavenger guild, composed of mainly meso‐carnivores, which plays essential roles in nutrient cycling, disease control, and other ecosystem functions (Beasley et al. 2015; DeVault et al. 2003). Given their reliance on carrion, these species are likely to respond to changes in carrion availability and land use, particularly pastoralism. Changes in scavenging behavior could have broader ecological consequences (Mateo‐Tomás et al. 2017), such as meso‐carnivore release (Saggiomo et al. 2021) or increased livestock predation, potentially escalating human–wildlife conflict and illegal poisoning (Nattrass and Conradie 2018). Despite the importance of these dynamics, the effects of pastoralism on terrestrial scavenger communities remain largely unexplored, especially in the Eastern Mediterranean, which differs from the Western Mediterranean in both its history of livestock husbandry and the composition of its scavenger guild (Arrondo et al. 2023; Cheylan 2008).

The main aim of this study is to investigate whether habitat type (pastoral or nonpastoral) differentially influences species presence, interactions, and carrion preferences (domestic versus native) among the East Mediterranean terrestrial scavenger guild, as an example for other ecosystems. The initial hypothesis is that there would be differences in both carrion preference and facultative scavenger species interactions as a result of the additional resources provided by livestock husbandry in natural habitats. To examine this hypothesis, an in situ field experiment was designed based on the placement of wild carrion (wild boar) and domestic carrion (cow) in pastoral and nonpastoral lands. This study addresses both practical conservation challenges associated with livestock husbandry management and theoretical considerations in nature conservation. From a theoretical standpoint, given the millennia‐long presence of pastoralism in the region's ecosystems, this study seeks to illustrate the role of humans, via cattle husbandry, in shaping large‐scale ecosystems and to address the conceptual question of whether pastoralism should be considered an integral part of the natural food web and environment. By examining pastoralism as a globally significant land use, this study advances our understanding of its potential to drive broad‐scale patterns in ecosystem functioning and food web organization.

## 2 Methods

### 2.1 Study Sites

As one of the main goals of our study was to describe the carrion preferences of both facultative and obligatory scavenging species and to understand how the effects of pastoralism alter these preferences, we needed to identify habitats in the eastern Mediterranean (specifically Israel) in which we could explicitly identify the two different types of land uses to be compared—pastoral and nonpastoral. Therefore, we looked for areas that meet the following three main conditions: 1. known presence of scavenging species; 2. natural habitats without predator culling and minimum human interference; and 3. distinct pastoral lands within the natural (nonpastoral) site.

**FIGURE 1:**
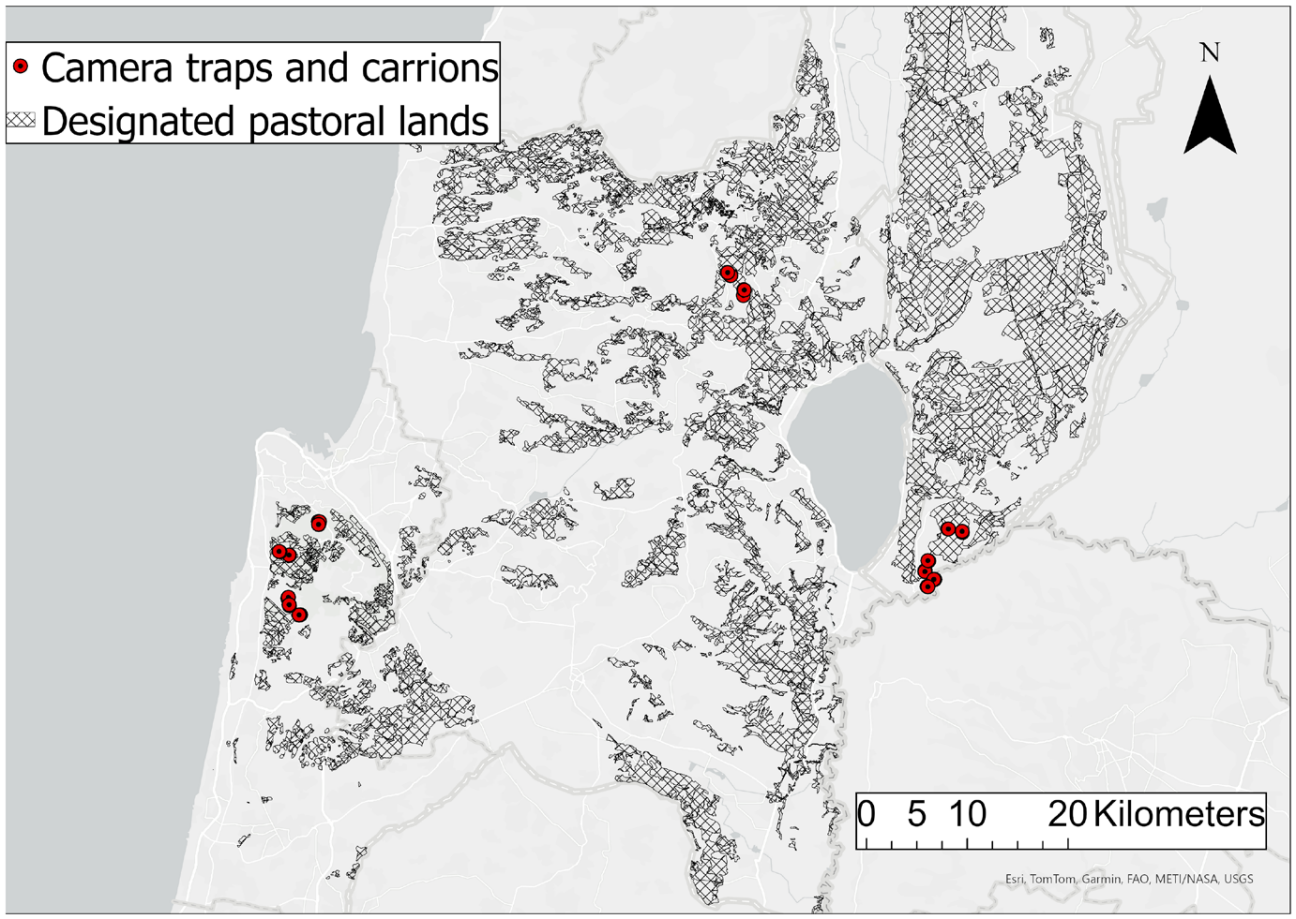
Study sites and camera trap locations.

To meet the first condition, we used data gathered by the Israel Nature and Parks Authority (INPA) composed of random and survey sightings of our species of interest: golden jackal ( *Canis aureus* ), red fox ( *Vulpes vulpes* ), and wild boar ( *Sus scrofa* ) along with the local apex predator (the wolf, *Canis lupus* ) and what is considered an apex scavenger, the striped hyena ( *Hyaena hyaena* ). In line with the second condition, we identified three suitable sites (Figure 1): the Carmel National Park, the Amud Nature Reserve, and the Meitzar Nature Reserve. The Carmel National Park, sized 120 km^2^, is characterized by a Mediterranean forest of oaks (*Quercus calliprinos*) and carob ( *Ceratonia siliqua* ) trees, or planted pine (*Pinus* spp.) forests at an altitude of mean ~400 m above sea level. There are no human settlements within the park, yet it is surrounded by some villages. The Amud Nature Reserve, sized 50 km^2^, is also characterized by a Mediterranean forest, at an altitude of ~600 m above sea level. This reserve is also surrounded by small villages and towns. The Meitzar Nature Reserve is at the southern slopes of the Golan Heights, and its habitats range from Mediterranean shrubs to park forest of different oak species. This reserve size is 30 km^2^ and its mean altitude is 300 m above sea level. To meet the third criterion, we used Israel's Ministry of Agriculture (IMOA) GIS layer of designated pastoral lands to identify pastoral and nonpastoral areas in regions of interest.

### 2.2 Sampling Design

**FIGURE 2:**
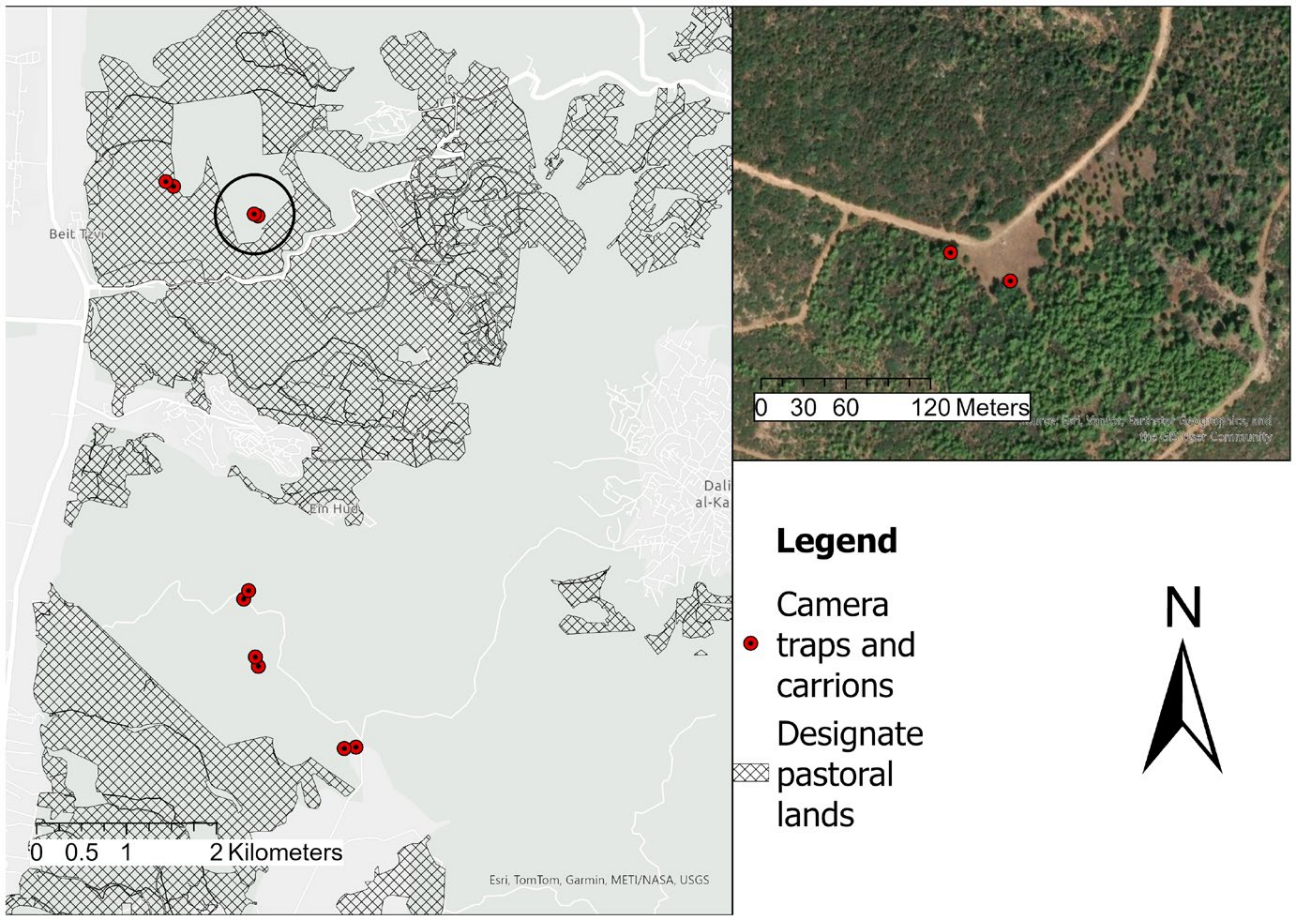
Crmel National Park study site and sampling units. The upper right panel shows a single sampling unit composed of carrion+CT (red dots).

In this study, we placed two types of carrions (Boar and Cow) in each habitat (pastoral and nonpastoral) and the carrions were monitored by camera traps (CT's). Each pair of CT's consists a Sampling Unit (SU). Each carrion type was placed 100 m from the other, in order to facilitate a choice selection for the scavenging species. The locations of each of the sampling units was at least 1 km afar to verify independency (Figure 2). In the Carmel site, we deployed a total of 14 CTs (8 in pastoral lands and 6 in nonpastoral lands). At the Amud site, we deployed a total of 8 CTs (4 in pastoral lands and 4 in nonpastoral lands), and in the Meitzar site, we deployed a total of 12 CT's (6 in pastoral lands and 6 in nonpastoral lands). The carrion along with accompanying CT's were deployed at two sampling campaigns—from march 2023 to May 2023. The carrions were “frozen fresh” and defrosted 24 h prior to placement. We used boar's leg or cow's leg obtained from legal hunting (boars) or dead cows supplied directly from farmers. The carrions were placed at a fortified metal cage tied with a metal wire to a tree or a rock. The use of this method has been chosen for ethical and practical reasons. Ethically, in Israel, hunters and farmers are legally required to remove carrion from the field upon discovery to minimize food subsidies. We wanted our study to adhere to this standard. Practically, we estimated that using these open cages would attract scavengers on one hand but would prevent them from completely depleting the carrion. As a result, the carrion parts persisted longer which provided for a larger dataset. This specifically made metal cage enabled scavengers to sense the carrion, yet it was almost impossible for them to feed on it. In this manner, it was possible for us to collect more data while also examine our specific research questions. We based our camera traps (CTs) with carrion locations near main trails in‐order to facilitate access.

CT's model was RECONYX hyperFire X and CT's were placed 4–8 m from the metal cage containing the carrion at a height of approximately 80 cm. When cameras detected motion, they captured a still image immediately, followed by a 30 s video. There was no delay between consecutive shots, and movement sensors were set to “high”. Cameras were retrieved after a maximum of 14 days working time. As discussed above, because the carcasses' legs were held in a metal cage, we assumed that the scent of the carcass would remain for this time period, even if the flesh is depleted. In this manner, we could collect data over a longer period without visiting the carcass site, which may alter the scavengers' behavior.

### 2.3 Focal Species

In this study, we will focus on three opportunistic facultative scavengers (Golden jackal, red fox, wild boar), the apex predator of the ecosystem, the gray wolf, and the only known mammalian obligatory scavenger, the striped hyena. Other possible scavenging mammals in the local ecosystem include common badger (*Melles melles*), stone marten ( *Martes foina* ), and the wild cat (*Fellis Silverstis*). Also present are medium‐ to small‐sized noncarnivore species such as the endangered mountain gazelles ( *Gazella gazella* ), the crested porcupine ( *Hystrix indica* ), the common rabbit ( *Lepus capensis* ), and more, though these species are not the focus of our study; they could serve as prey for facultative scavenging species.

### 2.4 Data Management

We first screened the cameras using a trained mega‐detector AI model (Beery et al. 2019) that differentiated between blank images/videos, as well as images with a person, vehicle, or wildlife. We set the confidence of the model to 20% to ensure relevant images were not incorrectly filtered out, and we manually inspected filtered results to validate the screening of the model. We then piped the output of the model to “Timelapse” (Greenberg 2020), software for additional screening of blank images. Next, for each remaining image and video file, we recorded the species, number of individuals, and sex of the individual(s) when possible. After tagging each file, a CSV file was compiled and exported to R version 2024.4.2 to conduct statistical analyses (Posit Team 2025). A camera trap event was considered as such if there was a time gap of at least 15 min between detections of the same species at the same CT.

### 2.5 Data Analysis

#### 2.5.1 Modeling Approach

##### 2.5.1.1 Effect of Pastoral Lands and Carrion Type on Camera Encounter Rate

To assess the differences in the carrion preferences of facultative scavengers, with respect to pastoral versus nonpastoral lands, we used a negative binomial generalized linear mixed model (GLMM) fitted for each individual species of interest in each individual camera trap. For these models, we created a normalized relative abundance index (nRAI) to account for inherent differences in species abundances. This nRAI was created as follows:

$$\text{nRAI}=\left(\mathrm{Ei}/\mathrm{Et}\right)/D\times 100$$

where *Ei* is an event of species *i* and *Et* is the total number of events in a sampling unit, and *D* is the number of days the camera was active. For example, if a fox was “captured” four times, and the total amount of photos at the same CT that operated for 10 days was 20 for all species combined, then the nRAI is [(4/20)/10] × 100 = 2.

As exploratory variables, we used the study site (i.e., Meitzar, Amud, Carmel) as a random factor and habitat type (pastoral versus nonpastoral) as a fixed effect and the nRAI as the structure of the. We chose a negative binomial model after preliminary examination of different families of distributions. All modeling was conducted using the R “lme4” package (Bates et al. 2015), along with the “SjPlot” package (Lüdecke 2023) for table summary and representation of model outputs. Model fit and diagnostics were examined with the “performance” package (Ludecke et al. 2020), using the functions “check_overdispersion” and “check_convergence”. To improve clarity in model interpretation, we present results using the incidence rate ratio (IRR), which quantifies the proportional change in the outcome associated with each predictor. We consider this approach more intuitive and communicative than reporting untransformed coefficients.

##### 2.5.1.2 Effect of Pastoral Lands on Species Interactions

To describe species interactions, we used piecewise structural equation modeling (PSEM) to analyze the number of CT events data. For this analysis, we used pooled data of each species across both carrion types, separated by land‐use (P vs. NP), as the main question we wanted to answer was what effect does the land‐use have on species interaction, regardless of carrion type. In this manner we were also able to reach an adequate sample size for this analysis. PSEM is a commonly used modeling framework for modeling species interactions (Lefcheck et al. 2016). The idea behind this process is to make an a priori hypothesis of the relationships between variables (species in our case) and then model the joint variation, composed of each “piece” of the model (hence—piecewise SEM) along with its direct and indirect effect. PSEM can also handle non‐normally distributed and correlated data which is very frequently the case in ecological research (Walsh 2019; Du et al. 2024). We partitioned the data by habitat type and created two identical PSEM models to describe the hypothesized species interactions, one for pastoral lands and one for nonpastoral lands. For each model we incorporated the study site (i.e., Meitzar, Amud, Carmel) as a random factor to account for sites variability. The structure of the PSEM model (i.e., species interactions) was based mainly on species body size and documented species behaviors or interactions. We generally assume that larger sized species or a higher trophic level of carnivores will have a negative impact on smaller species (like in the case of jackals on foxes) or lower trophic levels (like in the case of gray wolves and boars) (Table 1).

**TABLE 1:** Hypothesized PSEM components. The assumed species camera trap events in response to a “predictor” scavenging species.

Response species	Predictor species
Gray wolf	—
Wild boar	Wolves
Golden jackal	Wolves + boars
Red fox	Wolves + boars + jackals
Wild cats	Jackals + foxes

Individual “pieces” of the PSEM models (represented by rows in Table 1) were conducted using the R package “lme4” as GLMM's, with the number of CT events per species as the response variable and the days the camera was active as an offset variable and a negative binomial distribution. We examined model convergence and residual dispersion with the R package “performance”. To test the goodness of fit of the complete PSEM, we used a Chi‐square test. It should also be noted that the output of the PSEM analysis provides a standardized estimate which allows for the comparison of the effect of each pairwise species interaction. We manually and graphically plotted the results of the models instead of using the default path diagram provided by the R package.

## 3 Results

### 3.1 Sampling Effort and Encounter Rates (ERI)

During our camera trap campaigns, we operated 32 CTs (5 were excluded due to technical failures or malfunctions) for a total of 212 camera trap days and collected a total of 939 images with animals that were aggregated to 797 CT events when considering a minimal time difference of 15 min between the captured images of the same species. For a summary table of the number of CT events per species, refer to Appendix B. The most frequently recorded species was the wild boar with 323 CT events, followed by the golden jackal with 193 CT events and the red fox with 140 CT events. The only apex predator at our study site, the gray wolf, appeared in 10 CT events. All the other CT events were of other carnivores such as the stone marten, the feral cat, the common badger, or the only obligatory scavenger at our study site, the striped hyena, which appeared in only 8 CT events across only two locations. In terms of nRAI, the wild boar has the highest indices, while hyenas had the lowest index. We also documented noncarnivore species such as the mountain gazelle ( *Gazella gazella* ), the fallow deer ( *Dama mesopotamica* ), and the crested porcupine ( *Hystrix indica* ). These species were omitted from the analysis.

### 3.2 Carrion Preferences

**FIGURE 3:**
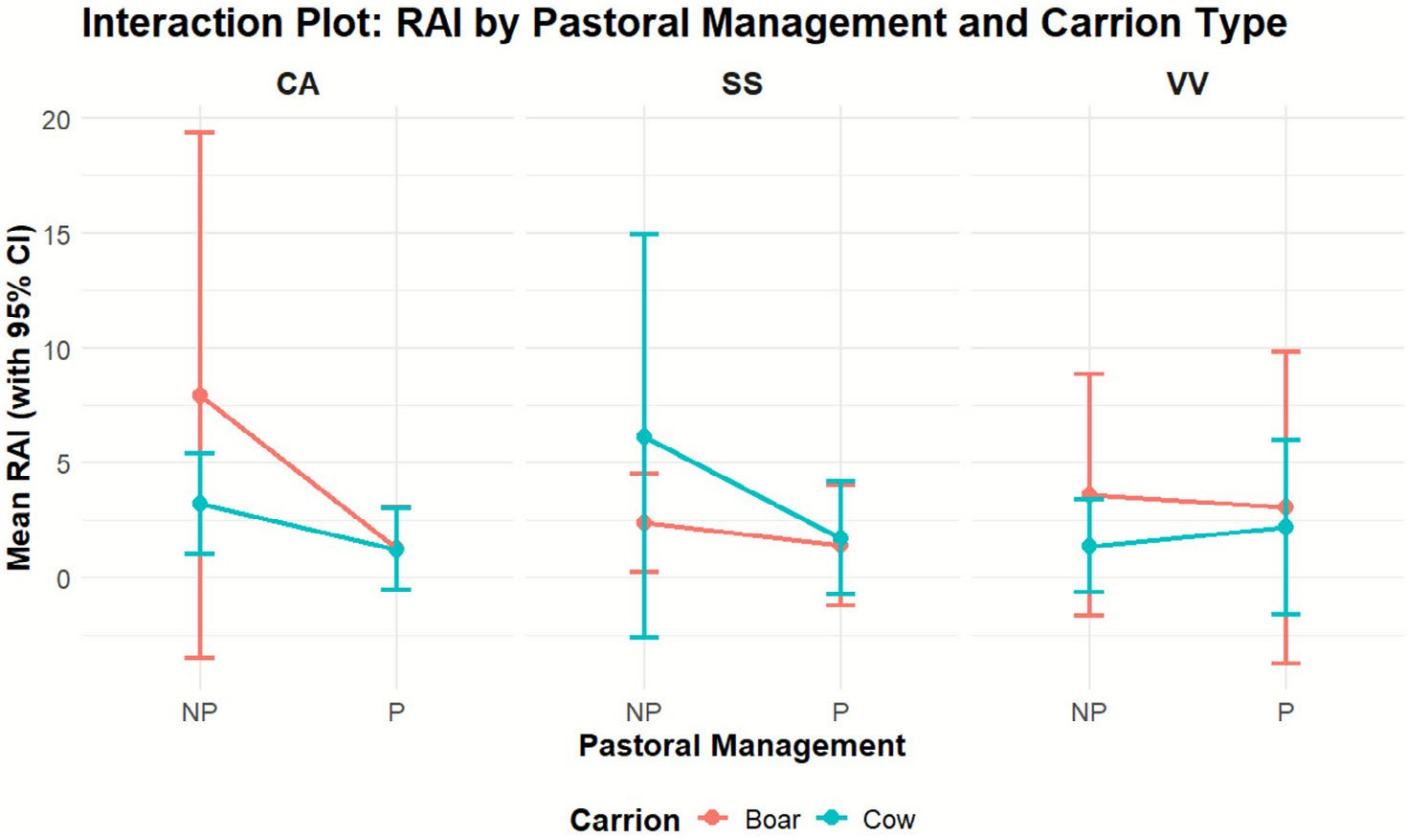
Mean nRAI of jackals (CA), boars (SS), and foxes (VV); significant difference in the nRAI of species (regardless of carrion type) is highlighted by *.

For the species for which we had an adequate sample size and representations throughout both habitat types and in all study sites (more than 10 events per group), we fitted GLMMs in order to examine incidence rate ratio (IRR) of facultative scavengers in response to land use (pastoral/nonpastoral) and carrion type—wild (boar) or domestic (cow) carrion (Figure 3, Table 2, Appendix C). For red foxes, a significant effect of land use was found (i.e., pastoral vs. nonpastoral) on the camera encounter rate of each carrion type. Nevertheless, we saw that generally there was a lower incidence rate ratio (IRR) at cow's carrion, in pastoral lands, while the interaction between these two variables led to a higher IRR of foxes.

**TABLE 2:** Models summary table. Incident rate ratio of species CT (and *p* values) events by habitat and carrion type.

Species	Habitat (pastoral relative to nonpastoral)	Carrion (cow relative to boar)	Interaction (Pastoral relative to nonpastoral* Cow relative to boar)
Foxes	−35%	−71%	+173%
Jackals	−84%***	+59%	+139%
Boars	−79%	+16%	+30%

* *p* < 0.05.

** *p* < 0.01.

*** *p* < 0.001.

Additionally, jackals showed a significant decrease of 84% in the IRR of CT events in pastoral lands when compared to nonpastoral lands (IRR = 0.16% *p* = 0.0.020). There were no significant interactions between models term yet; the IRR of a CT event on cows' carrion in pastoral lands was 139% higher. There was no variability captured between studies region and the in‐region variability was much more significant than between‐region variability. The marginal R^2^ of the model was 0.416.

We also evaluated how pastoral lands influenced the carrion preferences of wild boars. We found no significant effects, yet generally boars' IRR of CT events was higher at cow's carrion and lower at pastoral lands. For all species, we did not see any significant interactions between land use type and carrion's type.

### 3.3 Species Interactions

**FIGURE 4:**
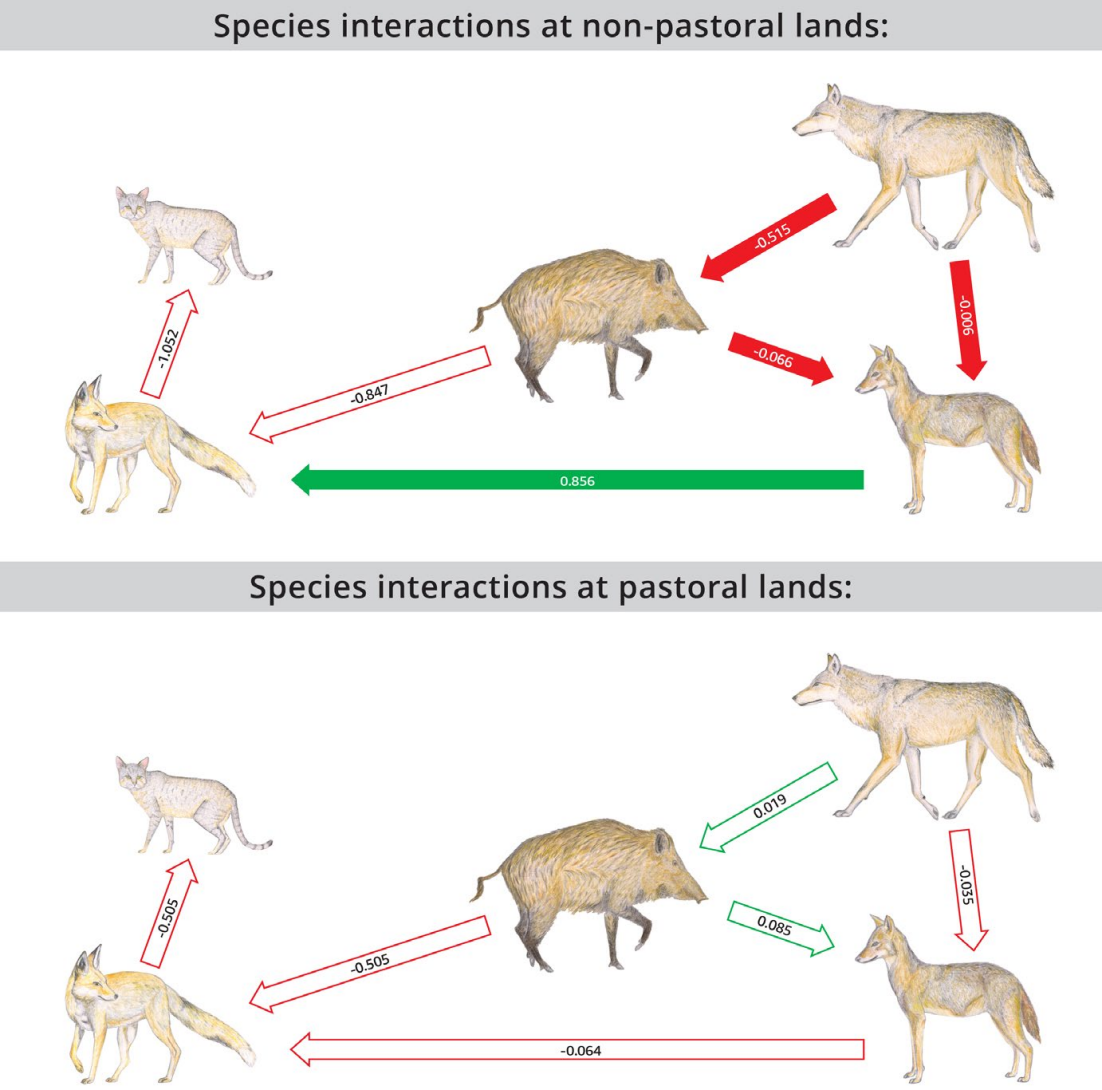
PSEM results. Filled arrows represent significant interaction, Red represent negative effect and green represent positive effect. Numbers are the standardized coefficients, reflecting comparable effect sizes.

We used two PSEM models to describe our data, one for pastoral lands and one for nonpastoral habitats with the pooled CT events of species, regardless of carrion type given a priori hypotheses of the structure of the interactions of the most dominant facultative scavenger species—jackals, boars, foxes, wild cats, and wolves. Both models fitted the data well (Pastoral: chi‐squared = 0.842, *p* = 0.839; Nonpastoral: chi‐squared = 2.795, *p* = 0.095). In nonpastoral lands, there were more negative and significant interactions among facultative scavenging mammals than in pastoral lands (Figure 4, Appendix A). It should also be stated that even though wolves approached near the carrion, there was no documentation of these species actually interacting with the metal cages in contradiction to other species.

## 4 Discussion

Our goal was to study the effect of pastoralism as a land use—on terrestrial facultative scavenger species in Mediterranean ecosystems and more specifically—aspects of carrion preferences and species interactions, disregarding the presence of cattle. To address these objectives, we examined two key ecological aspects of mammalian land use: (1) feeding ecology of scavenger species through “cafeteria” experiments in relation to habitat type (pastoral vs. nonpastoral) and carrion type (domestic vs. native) and (2) scavenger species interactions as a function of habitat type.

### 4.1 Effect of Pastoral Lands on Species Camera Trap Encounter Rate and Carrion Preferences

Regardless of carrion type, we saw that all the examined facultative scavengers had a lower IRR of CT events in pastoral land with only jackals exhibiting a significant effect (table 3). We propose that this finding could be attributed to the fact that on nonpastoral lands, naturally occurring carrions are much less abundant, and therefore facultative scavengers are more motivated to find and feed on carrions when they are available, as discussed in Vandersteen et al. (2023). The difference in CT events in pastoral versus nonpastoral lands may also be explained by the “resource dispersion theory” (Macdonald 1983; Macdonald and Johnson 2001). This theory describes how the spatial and temporal distribution of resources influences animal group size, territory size, and other ecological traits. We suggest that our results align with this theory, as the studied species demonstrated a differential response to the provided carrions. The fact that the IRR of CT events of the golden jackal—considered “eruptive” within the Israeli fauna, and considered by farmers as a highly damaging species (Shapira et al. 2024) – that may be affected by pastoralism, is an important outcome of this study and should be considered by conservation and wildlife agencies.

Another interesting finding of our study is the fact that albeit being insignificant, it was shown that the three main facultative scavenger species' IRR of CT events was higher in the combination of cow's carrion on pastoral lands. This can imply the fact that when given the opportunity in pastoral lands, these species will prefer to feed on cow's carrion rather than on boar's carrion. This could imply the effect these added food resources (i.e., cows' carrion) could have on the feeding behavior of scavenging species.

Regarding the less frequently observed species in our study, feral cats have been shown to scavenge in Europe primarily during winter (Vandersteen et al. 2023). In contrast, our study was conducted in spring, under warmer climatic conditions, which may explain their low camera trap encounter rate and the discrepancies between our findings and those of Krofel et al. (2021).

In contrast to our sporadic documentation of the ecosystem apex predator, the gray wolf, Ciucci et al. (2020) observed a behavioral shift in this species' scavenging behavior, noting that wolves in pastoral lands preyed less, scavenged more, and relied more heavily on cattle carrion compared to wild boars, roe deer ( *Capreolus capreolus* ), and more. Their general absence in our study could be attributed to their general reliance on self‐made kills in this region (Wikenros et al. 2023) or because they are generally scarce in the study areas. We also saw that even when wolves did approach carcasses, they tended to do so cautiously and with vigilance.

### 4.2 Species Interactions and Habitat Type

The PSEM demonstrated that in nonpastoral lands, wild boars’ function to some extent as “top scavengers”. This could be seen by their functioning ecological traits, aligned with those discussed in Méndez et al. (2024). Wild boars were observed at most carrion sites, in large numbers, with no co‐occurring species. The only trait missing to define wild boars as “Apex scavangers” was the “together” trait, as wild boars generally consumed carrion parts to their full and left no parts for other, smaller species. Wallach et al. (2015) defined apex predators by their top‐down influence on community structure, and our findings suggest that boars similarly shape scavenger communities in ecosystems lacking true apex predators or ecosystems that have low abundances of apex scavengers. Wild boars’ dominance was especially evident in nonpastoral (more pristine) habitats, where significant negative interactions with smaller carnivores were observed, likely due to intense competition over limited food resources. Notably, boars were negatively affected only by the presence of gray wolves (even if wolves CT events have been sparse), highlighting the role that a single species of apex predator can play in the ecosystem (Newsome et al. 2017; Ripple et al. 2014). This dynamic is echoed by Focardi et al. (2017), who reported competition between boars and wolves over wolf‐killed carcasses in Italy's Northern Apennines, or by Olea et al. (2022) who demonstrated similar concepts with avian scavenging communities, as opposed to terrestrial ones. To our knowledge, ours is the first documented case of wild boars acting as top scavengers in natural, yet humanly influenced habitats.

One of the most compelling findings of our study is the stark contrast in species interactions between pastoral and nonpastoral lands, as revealed by the PSEM. In pastoral lands, no significant interactions between scavenger species were detected, suggesting a more diffuse and less competitive community. In contrast, nonpastoral lands exhibited a more structured and competitive scavenger network, with predominantly negative interactions. These patterns align with previous work showing that anthropogenic food subsidies can alter species interactions and ecological processes (Oro et al. 2013; Moreno‐Opo and Margalida 2019). Our findings also support those of Selva and Fortuna (2007), who demonstrated that both carrion type and habitat structure influence the complexity of scavenger communities.

A deeper look at the standardized regression coefficients reveals key shifts in interaction patterns across land types (table 3). For instance, wolves had no significant effect on boar ERI in pastoral lands, while the effect was significantly negative in nonpastoral lands—possibly reflecting weaker competition or predation pressure in areas where livestock carrion is more abundant. However, due to the low number of wolf CT events in our study, interpretations regarding their influence should be made with caution.

A similar shift was observed in the interaction between boars and jackals. Additionally, jackals positively affected fox ERI in nonpastoral lands, an interaction not seen in pastoral lands. While the cause of this discrepancy is unclear, it may reflect heightened competition in carrion‐limited environments. Still, sample size limitations may also contribute and should be addressed in future research. We further observed that in nonpastoral lands, wolves negatively influenced jackal ERI, and jackals in turn negatively influenced fox ERI, consistent with mesopredator suppression dynamics described by Newsome et al. (2017) and the meso‐carnivore release hypothesis (Prugh et al. 2009). Prior research has similarly shown that jackals typically displace foxes (Scheinin et al. 2006; Jaklič and Potočnik 2022; Kapota et al. 2016). In summary, we propose that the intensified negative interactions observed in nonpastoral lands are driven by lower resource availability and stronger competition and exhibit “natural” processes. In contrast, the expected greater availability of food in pastoral lands may facilitate co‐sympatry and reduce interspecific conflict (Lacombe et al. 2024).

Methodically, it should be stated that camera trap studies have inherent limitations arising from possible spatial biases or under‐sampling. Furthermore, as common in such studies, camera trap data reflect encounter rates rather than actual population abundance. For species that are difficult to identify individually, estimating total abundance would require individual recognition techniques, potentially leveraging emerging AI technologies (Fergus et al. 2024). However, the robustness and consistency of our findings across a broad experimental setup, in three unique study sites, strengthen the validity of our conclusions. Furthermore, while our study focused on Mediterranean systems, the patterns observed here may apply to other ecosystems where pastoralism or similar land uses provide predictable food subsidies to vertebrate scavengers. This suggests a broader pattern of altered species interactions in human‐dominated landscapes.

## 5 Conclusions

First, there are some caveats to our study that need to be considered. (1) The sampling size (in terms of repetitions) in this study was limited, as is usually the case in broad scale ecological field research; (2) The number of CT events for some species was sparse (like wolves); and (3) This study was limited to Mediterranean habitats; and (4) Our field method is novel and could possibly affect results. On one hand, our results should therefore be handled carefully, but on the other hand, even with these limitations, we observed clear, significant, and consistent patterns that could apply to other ecosystems as well.

Our research demonstrated differences in the nRAI of scavenger species in pastoral versus nonpastoral habitats and a significantly lower IRR of CT events of jackals on pastoral lands when compared to nonpastoral lands. We also showed that on pastoral lands, species interactions are considerably different than those observed in pristine nonpastoral lands, with wild boars acting as apex scavengers only in the latter. Therefore, we argue that pastoralism as a land use can have a strong effect on the natural food‐web structure—or rather its lack of structure, as demonstrated in our east Mediterranean study sites. This, in turn, could have a cascading effect on other aspects of nature conservation, such as a contribution to the eruption of meso‐carnivores, which leads to a higher predation pressure on smaller species. Given that pastoralism has been an integral part of East Mediterranean ecosystems for over 10,000 years, it is challenging to determine the optimal management regime of natural habitats—and whether pastoralism is “good” or “bad” for species and food‐web conservation. Thus, we conclude that nature conservation agencies managing pastoral lands in protected areas (e.g., nature reserves) should adopt a balanced approach to pastoralism, as demonstrated in Zelnik et al. (2021) and in concordance with the intermediate disturbance theory (Connell 1978). In the context of human influence on natural environments—such as pastoral lands—and considering the global scale of this land use, our results show that pastoralism as a land use can significantly impact natural food webs, as demonstrated in our study of facultative scavenger guilds. This, in turn, should be accounted for when considering pastoralism in protected, natural habitats.

## Author Contributions

**Ori Shapira:** conceptualization (lead), data curation (lead), formal analysis (lead), methodology (equal), project administration (lead), software (lead), visualization (lead), writing – original draft (lead). **Ido Izhaki:** conceptualization (equal), methodology (equal), supervision (equal), validation (equal), writing – review and editing (equal). **Shiri Zemah‐Shamir:** supervision (equal), validation (equal), writing – review and editing (equal). **Dan Malkinson:** conceptualization (equal), methodology (equal), software (equal), supervision (equal), validation (equal), writing – review and editing (equal).

## Conflicts of Interest

The authors declare no conflicts of interest.

## Supporting information


**Data S1:** ece372839‐sup‐0001‐Appendix.docx.

**Data S2:** ece372839‐sup‐0002‐CT‐sum‐CP29.08.23‐Correct.csv.

## Data Availability

Data is added to files attached to this submission.
