# Supplementary material for: How Pastoral Are Pastoral Landscapes? Scavenger Assemblage Structure in Human‐Dominated Landscapes: A Case Study From Mediterranean Pastures
Source: Ecol Evol. 2026 Jan 14;16(1):e72839. doi: 10.1002/ece3.72839 (PMC12800921; doi:10.1002/ece3.72839)

**Appendix A:**

Table 3: Summary table of standadized regression coefficients of both PSEM models Pastoal and non-pastoral (*P < 0.05). Carrion type pooled.

| *Species pair* | *Pastoral* | *Non-pastoral* |
| --- | --- | --- |
| *Wolves-boars* | 0.019 | -0.515* |
| *Boars-jackals* | 0.085 | -0.006* |
| *Boars-foxes* | -0.505 | -0.847 |
| *Jackal-foxes* | -0.604 | 0.856* |
| *Foxes-wild cats* | -0.505 | -1.052 |
| *Wolves- jackals* | -0.035 | -0.06* |

**Appendix B – total number of CT events per species used for the analysis:**


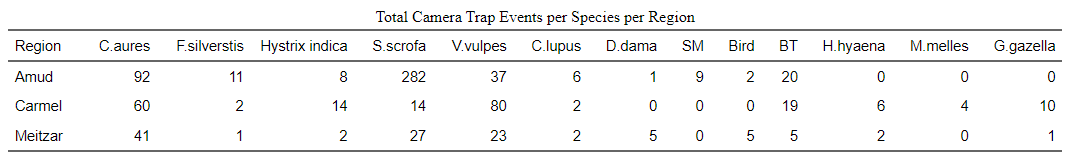


**Appendix C – Carrion preference model outputs (GLMM):**

**Foxes:**


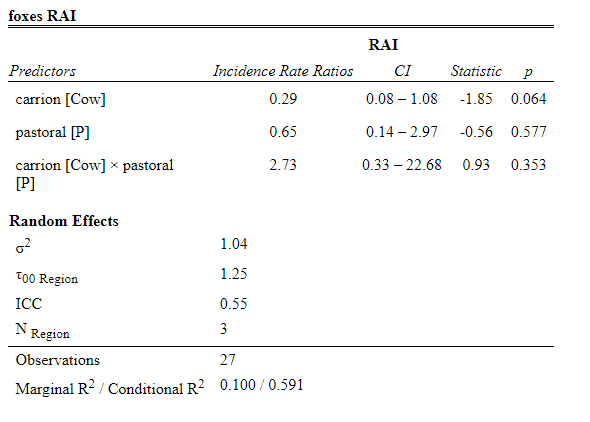


**Jackals:**


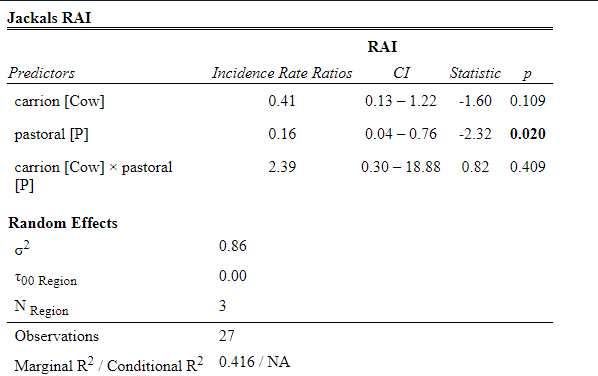


**Boars:**


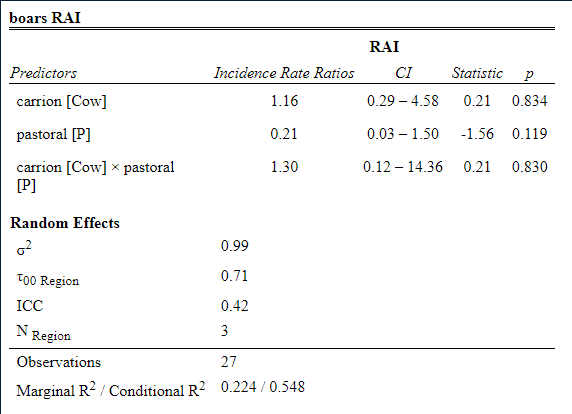

Supplement: Supplementary file 1 — Data S1: ece372839‐sup‐0001‐Appendix.docx. [file ECE3-16-e72839-s002.docx]
